# Psychological Well-Being of Young Athletes with Physical Disabilities: A Systematic Review

**DOI:** 10.3390/bs14090822

**Published:** 2024-09-14

**Authors:** Olatz Zabala-Dominguez, Yolanda Lázaro Fernández, Isabel Rubio Florido, Jurgi Olasagasti-Ibargoien

**Affiliations:** 1 Deusto Sports and Society, Department of Physical Activity and Sports, Faculty of Education and Sport, University of Deusto, 48007 Bilbao, Spain; yolanda.lazaro@deusto.es (Y.L.F.); irubio@deusto.es (I.R.F.); 2 Health, Physical Activity and Sports Science Laboratory, Department of Physical Activity and Sports, Faculty of Education and Sport, University of Deusto, 48007 Bilbao, Spain; jurgi.olasagasti@deusto.es

**Keywords:** psychological well-being, physical disability, athletes, sports

## Abstract

Currently, young people with disabilities practice less sport than people without disabilities, which is a risk to their health and affects their well-being. The aim of this research was to identify the specific dimensions and tools used to measure psychological well-being (PWB) in young athletes with physical disabilities and to analyze the influence of sport. A systematic review was conducted using PUBMED/MEDLINE, Web of Science, Scopus, and Sportdiscus databases, and the search was completed without limitation to any specific year, including results up to and including 29 May, 2023. A total of 2112 articles were obtained in the initial search. Eligibility criteria were: (i) athletes with a physical disability, (ii) performing adapted and non-adapted physical activity and/or sport, (iii) the study aimed to analyse well-being, and (iv) outcomes of psychological well-being variables were identified. Exclusion criteria were (i) people with injury or transient difficulty, (ii) rehabilitative physical activity, and (iii) the results did not show any psychological well-being variables or mentions. This review identified that the most used dimension was mastery of the environment, together with the autonomy. All studies showed a positive correlation between sport practice and psychological well-being. Five measurement tools were identified knowing the state of psychological well-being of young athletes with disabilities is essential to promote successful participation in sports activities.

## 1. Introduction

A significant part of the general population is composed of people with disabilities; worldwide, the rate stands at 1 billion people [1]. Estimates by the United Nations Children’s Fund [2] indicate that the number of children and adolescents under 18 years of age with disabilities is 240 million worldwide. Physical disability is defined as a motor impairment that limits to a greater or lesser extent daily activities of daily living [3]. Young people with disabilities have to face obstacles and barriers that hinder their inclusion in society. Among the most frequent barriers, we find personal barriers caused by the disability itself and health status; social barriers, caused by discrimination and stigmatization; psychological barriers due to low levels of self-esteem and lack of motivation; and, finally, physical barriers due to lack of accessibility and adapted transportation and equipment [4,5].

Due to its characteristics, sport is a key tool in the fight for equality, the removal of barriers, and social transformation. It is a booming phenomenon in today’s society due to the multiple benefits it provides on a physical, psychological, and social level [6]. The practice of sports by people with disabilities is called adapted sport and refers to sports that are adapted to the specific needs of each disability [7]. In today’s society, the progress of adapted sports and interest is increasing, a result of which is the increase in the number of publications in recent years with the term “adapted sport” is exponential [8]. These benefits are even greater in people with disabilities, contributing to improved functional independence, improved physical capacity, greater self-confidence, improved mood, reduced stress and anxiety, and better relationships with others [5,9]. Despite this, people with disabilities practice less sport than people without disabilities. Studies indicate that they are between 16% and 64% less likely to meet the recommended minimums and consequently have a higher risk of suffering from sedentary-related health diseases affecting their well-being [10,11].

Studies have shown that sports practice has clear psychological benefits improving well-being in the general population [12]. Well-being is analyzed from two perspectives: hedonic well-being and eudamonic well-being. Hedonic well-being, known as subjective well-being (SW), is composed of affective-emotional and cognitive aspects and is used to evaluate the level of life satisfaction through positive and negative emotions and experiences of the human being. On the other hand, eudamonic well-being, known in turn as psychological well-being (PW), is associated with experience functional ability, and social mastery [13,14]. Carol Ryff defined PWB through her multidimensional theory and defined six dimensions that comprise it: autonomy (1): sense of self-determination, personal independence and self-regulation; mastery of the environment (2): ability to be effective with respect to managing life situations; personal growth (3): continuous development of one’s own potential; social bonds (4): the importance of positive attachments and relationships with others; projects (5): beliefs and goals that provide meaning to life; and acceptance (6): a positive appreciation of oneself [15,16]. To the authors, PW and SW are two different, but complementary, perspectives, and it is recommended to analyze both to obtain more information [1,17,18]. This is shown by the number of studies that analyze factors such as mood, quality of life (QOL), anxiety, self-esteem, environment, self-perception, social relationships, psychological disorders, and depression, together with PWB dimensions, to show a more global view [19,20,21,22].

As mentioned above, QOL, which, according to the authors, reflects the individual’s perception of life satisfaction, is a balance between expectations and achievements [23]. Therefore, in the literature, we found articles that have analyzed QoL to determine the well-being of athletes with physical disabilities [24,25,26,27].

Therefore, it is crucial to investigate the psychological well-being of athletes with disabilities in order to improve their support and optimize their sporting performance.

The objective of this research is to identify the specific dimensions and tools used to measure PWB in young athletes with physical disabilities to analyze the influence of sport and identify what other factors have a direct influence on these athletes’ PWB, with the aim of summarizing and providing a starting point for future research. For these reasons, in this paper, we present the results of a systematic review of the literature on psychological well-being in athletes with physical disabilities, with the purpose of analyzing the PWB of young athletes with physical disabilities.

## 2. Materials and Methods

### 2.1. Elegibility Criteria

No discrimination by country or race, age, or gender was made in order to obtain all possible research. Research articles published in English and/or Spanish were filtered.

The eligibility criteria used for inclusion were: (i) athletes with physical disabilities, (ii) perform physical activity and/or sport both adapted and non-adapted, (iii) the study aims to analyze well-being, and (iv) outcomes of psychological well-being variables are identified. The exclusion criteria applied to the research were the following: (i) people with an injury (not identified as a disability, such as people with a sprain), injuries or situations of difficulty for physical activity of a determined or transitory duration, (ii) physical activity for rehabilitation because it was considered a different activity objective, and (iii) the results did not show any variable or mention of psychological well-being. The studies were grouped according to the different types of psychological factors evaluated.

### 2.2. Information Sources

Pubmed/Medline, Web of Science (WOS), Scopus, and Sportdiscus were used as databases to perform a structured search. Using these high-quality databases, the search was completed without limitation to any specific year, and results were included up to and including 29 May 2023.

### 2.3. Search Strategies

Articles were retrieved from the electronic databases using different search strategies (Table 1), with such terms having to appear in the title or abstract. Articles considered to be of relevance in this field of activity were obtained using the snowball strategy linked to this equation.

### 2.4. Section Process

In addition, all relevant studies were found by reviewing the titles and abstracts of the articles in the databases and the results of the bibliographic searches. These articles were considered potentially relevant, and their compliance with the inclusion criteria was analyzed for the final analysis. In addition, the reference sections of all articles found were examined and all titles and abstracts obtained were cross-checked in order to detect possible duplicates or lack of actual studies on the topic. Titles and abstracts were also selected for further full-text review. The search for previous studies was performed separately by two different authors (O.Z.-D. and J.O.-I.), and possible discrepancies were discussed with a third author (Y.L.F.).

### 2.5. Data Collection

These results were grouped, on the one hand, those that had the objective of PWB and, on the other hand, those that analyzed PWB within general well-being. In each study, the dimensions used, other factors of well-being assessment and their relationship with PWB, the tools used, the profile of the subjects, and the results of the studies were identified.

Data extraction was performed manually by two independent reviewers (Reviewer 1 and Reviewer 2) using a standardized extraction form (see Table 2). Each reviewer independently reviewed the included studies and performed data extraction in a systematic manner. Discrepancies that arose during the extraction process were resolved by discussion and consensus among the reviewers. If no agreement was reached, a third reviewer (Reviewer 3), an expert in the field, was consulted to resolve the divergence. The entire data-extraction process was performed manually without the use of software or automation tools. The reviewers carefully transcribed the data into the standardized form, thoroughly reviewing each publication.

Data processing was structured in tables that synthesized the information according to the objectives of the manuscript (Table 2). A tabular format was used to identify the different tools used (Table 3) and to facilitate understanding of the domains analyzed in the studies (Table 4).

The authors registered this systematic review in OSF Registries. Ol Zabala-Domínguez. (27 July 2024) Psychological well-being in young athletes with physical disa-bilities: A systematic review. Retrieved from osf.io/8dkry (accessed on 25 July 2024).

### 2.6. Data Items

In this study, we did not collect quantitative data directly but focused on identifying and analyzing the tools used to measure psychological well-being (PWB) and the psychological factors assessed in the studies reviewed (Table 3). For this purpose, the dimensions or factors that allowed the identification of the instrumental part of each article were examined in order to determine how each study approached the measurement of these constructs. This analysis facilitated the understanding of the different methodological approaches used to assess PWB in the various studies included in this study.

### 2.7. Study Risk of Bias Assessment

Two authors (O.Z.-D. and J.O.-I.) assessed quality in terms of the methodology used, along with any risk of bias using the RoB 2 tool (Cochrane Collaboration, London, United Kingdom) [28], which is an updated version for assessing the risk of bias in randomized controlled trials. The RoB 2 tool allows a structured assessment of the risk of bias in five key domains: bias arising from the randomization process, bias due to deviations from the intended interventions, bias due to missing outcome data, bias in outcome measurement, and bias in the selection of the reported outcome. In case of lack of consensus among the reviewers, we submitted for assessment by a third party (Y.L.F.), following the protocol for study search and selection [29]. All points of the protocol were conducted: Verify whether the selected studies met the eligibility criteria. Compare all the papers found with each other to determine whether or not the studies overlapped: (A) The search results were combined by identifying the DOI of each article; in the absence of a DOI, a unique identifier was assigned to each article. (B) All searches of a database were summarized, and duplicates were eliminated using the DOI, in a first screening. (C) In a second screening, articles were sorted by title, and publication data (title, authors, journal, year, issue, and/or volume) were compared to eliminate duplicates. (D) All identified full texts were recruited for analysis.

The study selection process was conducted: Study selection was conducted according to the inclusion and exclusion criteria. (E) The decision to include a study was made in the favorable case of two investigators (O.Z.-D. and J.O.-I.). If a controversy in opinions arose, a third investigator (I.R.F.) intervened to make a final decision, following point 4 of the protocol, which established the inclusion criteria in case of disagreement, and point 5, which detailed the reasons for exclusion for discarded studies.

After inclusion of the articles, the studies were evaluated and quantified in relation to the risk of bias, using the RoB 2 tool procedure. This evaluation exhaustively considered each of the dimensions proposed by the tool.

## 3. Results

### 3.1. Search Process

**Figure 1:**
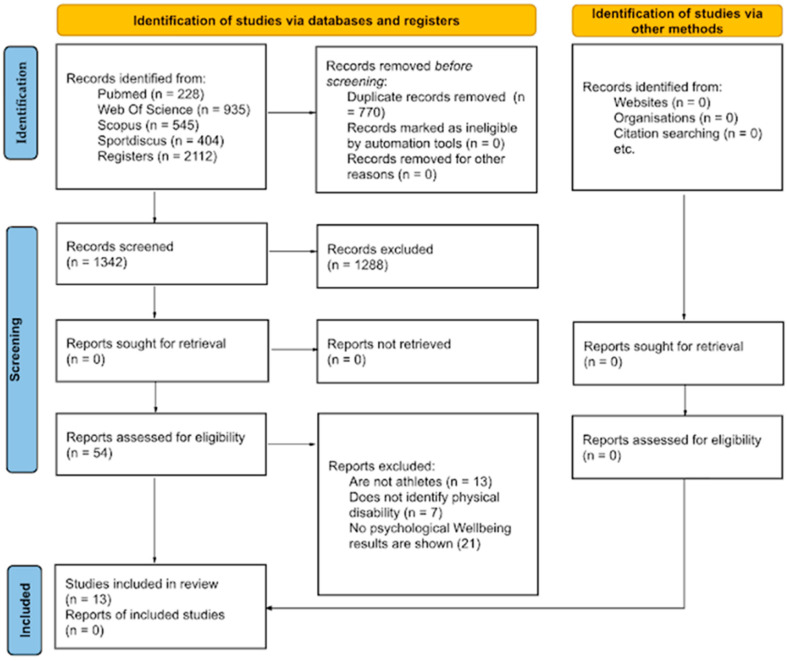
PRISMA flow diagram for study selection.

Of the 2112 articles found after searching different databases, only 13 were identified as meeting all of the inclusion criteria for the purposes of the systematic review, which are presented in a flow diagram [30] (Figure 1). Of these 2112 articles, 770 were eliminated because they were duplicates. Of the remaining 1342 articles, 1288 were eliminated after examination of titles or abstracts. Of the 54 full-text articles whose eligibility was assessed, an additional 41 papers were discarded because they were not athletes (n = 13), did not identify a physical disability (n = 7), or did not show results on psychological well-being (n = 21). Thus, the present systematic review included 13 studies [19,22,24,25,26,31,32,33,34,35,36,37,38]. All selected articles were found to have a low risk of bias after applying the RoB 2 assessment, too.

### 3.2. Data Extraction and Synthesis

In this section, Table 2 describes the studies analyzed, specifying their objective, the characteristics of the participants with the number of participants, age mean and the sport modality they practice, the specific PWB factors analyzed, other complementary factors, and the main results in alphabetical order.

**Table 2:** Main results.

Author/Year	Objective	Participants, Age Mean, and Sport Modality	Specific Factors of the PWB	Other Factors	Results
Goraczko et al., (2021) [31]	Analyzing self-efficacy and quality of life in athletes with physical disabilities	Athletes with physical disabilities Age mean: 39.5 (n = 16) Not specified	Psychological dimension of QOL	General Self-Efficacy Scale	Athletes who practice more sports have a higher PWB
Calheiros et al., (2020) [24]	To analyze the relationship between QoL and lifestyle in athletes with physical disabilities	Athletes with physical disabilities (n = 150) Age mean: 35.07 ± 9.45 Wheelchair Handball	Psychological dimension of VC	Not applicable	The greater the sport practice, the greater the PWB
Campbell, (1995) [19]	To analyze the differences in PWB in athletes with congenital and acquired physical disabilities	Athletes with congenital disability (n = 50) Athletes with an acquired disability (n = 43) Age mean: 25.9 (>17 years) Wheelchair sports	Environmental dominance (2)	Mental strength Trait anxiety Self-esteem	Athletes with acquired disability show higher PWB, have higher levels of mastery.
Campbell and Jones, (1994) [32]	To analyze the PWB in athletes and non-athletes and to analyze according to the level of according to the level of competition	Athletes with disabilities (n = 72) Non-athletes with disabilities (n = 29) Age mean: 32 (>17 years) Wheelchair sports	Environmental dominance (2)	Mental strength Trait anxiety Self-esteem	Sportsmen have higher mastery than non-athletes. The higher the level of competition, the higher the mastery
Nemček et al., (2020) [33]	To analyze the subjective perception of QoL in subjects with physical disabilities, athletes and non-athletes	Competitive athletes (n = 26) Recreational athletes (n = 45) Non-sportsmen (n = 59) Age mean: 24.4 ± 1.9 Boccia	Psychological dimension	Subjective quality of life	There is no difference in PWB between athletes and non-athletes
Jooste and Kubayi (2018) [34]	To analyze the levels of PWB shown by athletes with physical disability in Wheelchair Basketball	Athletes with disability (n = 16) Age mean: 32.13 ± 6.62 Wheelchair Basketball	(1) Autonomy (2) Mastery of the environment (3) Personal growth (4) Positive relationships (5) Life purpose (6) Self-acceptance	Subjective Vitality	Athletes show positive PWB values. The most valued dimensions: Personal growth (3) and Acceptance (6).
Yazicioglu et al., (2012) [26]	To compare the levels of QoL and Satisfaction with life between athletes and non-athletes with physical disabilities	Athletes (n = 30) Non-athletes (n = 30) Age: >18 years Not specified	Psychological dimension of QOL	Satisfaction with life	Athletes have higher levels of PWB
Medina et al., (2013) [35]	To analyze the relationship between PWB and type of sport practice in people with physical disability of neurological origin	Competitive sport group (n = 40) Age mean: 37.6 ± 15.6 Wheelchair Basketball, Tennis, Athletics and Boccia	Self-control/Mastery of the environment (2)	Not applicable	Athletes present higher levels of PWB and lower levels of anxiety and depression.
Yazici Gulay et al., (2022) [26]	To analyze the QOL of athletes and non-athletes with physical disabilities	Non-athletes (n = 19) Athletes: (n = 17) Boccia (n = 9) Wheelchair Basketball (n = 8) Age mean: 31.57 ± 10.15	Psychological dimension of QOL	Mobility Index Functional Independence Measurement Trunk impairment scale	Athletes have a higher psychological domain than non-athletes, and among sports, Boccia players have higher levels than wheelchair basketball players.
Zelenka et al., (2017) [22]	Analizar el efecto del Rugby en silla de ruedas sobre la CV de personas con lesión medular	Non-sportsmen (n = 16) Athletes (n = 20) Age mean: 32.5 ± 8.46 Wheelchair rugby	Psychological dimension of QoL	Not applicable	No difference between athletes and non-athletes
Trujillo Santana et al., (2022) [36]	Describe the PWB of high-performance athletes with and without disabilities	Athletes without disabilities (n = 31) Athletes with disability (n = 34) Age mean: 19.71 ± 1.96 Not specified	(2) Mastery of the environment Social links Projects Acceptance	Mental strength Subjective Vitality	Athletes show positive PWB values
Ciampolini et al., (2018) [37]	To compare the perception of QOL of Boccia, athletics, and wheelchair tennis athletes	Boccia (n = 41) Athletics (n = 14) Wheelchair Tennis (n = 31) Age mean: 39.35 ± 11.57	Psychological dimension of VC	Not applicable	Athletes have higher PWB than Boccia or wheelchair tennis players.
Nowak et al., (2022) [25]	To analyze the relationship between QoL and health satisfaction with the sport level of athletes with physical disabilities	Amateur athletes (n = 126) Professional athletes (n = 66) Age mean: 34.48 ± 8.79 Wheelchair basketball Wheelchair rugby Rowing Individual sports	Psychological dimension of QOL	Not applicable	Amateur athletes have higher CVs and satisfaction than competitive athletes. Among sport types, wheelchair basketball athletes have higher satisfaction and CV than rugby and rowing players.

### 3.3. Measurement Tools and Specific Dimensions Used to Measure PWB

In the review, we identified five and an adaptation-specific tool that analyzes PWB in athletes with physical disabilities (Table 3).

**Table 3:** Factors and tools.

PWB Factors Analyzed	PWB Tool (Items and Reliability)	Other Factors Analyzed (Those That Are Related to PWB)	Reference
Autonomy (1)	Psychological Well-Being Scale (PWBS) [38]	Subjective Vitality Scale (SVS) [39]	[34]
Mastery of the environment (2)			
Personal growth (3)			
Social bonds (4)			
Projects (5)			
Acceptance (6)			
Mastery of the environment (2)	Psychological Wellbeing Scale BIEPS [40]	Mental Toughness Inventory (MTI) [41]	[36]
Acceptance (6)			
Social bonds (4)			
Projects (5)			
Mastery of the environment/self-control (2)	Psychological well-being index (PWBI) [42]	Not applicable	[35]
Mastery of the environment (2)	Mastery [43]	Profile of Mood States (POMS) [44]	[19,32]
		State-Trait Anxiety Inventory (STAI) [45]	
		Self-Esteem Scale [46]	
Psychological dimension (CVd2)	World Health Organization Quality of Life BREF (WHOQoL-BREF) [47]	Not applicable	[22,24,25,38]
		Satisfaction with life [48]	[26]
		Subjective quality of life [49]	[33]
		Self-efficacy [50]	[31]
	World Health Organization Quality of Life Disability (WHOQOL-DIS) [51]	Rivermead Mobility Index (RMI) [52] Measurement of Functional Independence (MIF) [53] Scale trunk impairment (TIS) [54]	[26]

These were the dimensions most used to analyze the influence of sport on the psychological well-being of athletes with physical disabilities.

Regarding the studies whose main objective is to analyze psychological well-being (Table 4) without considering the studies that analyze the PWB in its second dimension of the CV (CVd2), we will analyze the dimensions based on Ryff’s multidimensional theory. Among the six dimensions identified, the domain of the environment is the dimension most used by five authors (Table 4).

**Table 4:** Studies included the objective of analyzing psychological well-being.

Domains	Number of Authors	Cites
Autonomy (1)	1	[34]
Mastery of the environment (2)	5	[19,32,34,35,36]
Personal growth (3)	1	[34]
Social bonds (4)	2	[34,36]
Projects (5)	2	[34,36]
Acceptance (6)	2	[34,36]

## 4. Discussion

The study has identified five tools for measuring psychological well-being, with them being the most analyzed dimensions.

Regarding measurement tools, the Psychological Well-Being Scale (PWBS) tool analyzes the six dimensions of Ryff’s multidimensional model: autonomy, mastery of the environment, personal growth, social ties, projects, and acceptance [38]. It is a complete scale and consists of 18 items. It is an adequate tool and meets the reliability criteria; its Cronbach’s index is between 0.81 to 088. It has recently been confirmed that it is an adequate tool [55].

Another specific tool based on Ryff’s multidimensional model is the Psychological Well-Being Scale (BIEPS) [40]. This scale measures four of the six dimensions: mastery, acceptance, social bonds, and project. It consists of 13 items, and, despite having a low reliability of 0.59, it is close to the values of the original scale, showing relevant and novel results. The measurement of PWB in teachers and hospital patients has been analyzed in non-sport contexts recently [56]. The authors of the scale developed a subsequent scale aimed at adults BIEPS-A [57], where it replaces the acceptance dimension with autonomy, has the same number of items, and may offer more relevant results in people with physical disabilities. Currently, this scale has been used in people without physical disabilities with a reliability of 0.70 in Cronbach’s Alpha coefficient. It has recently been used in adults in a Mexican sample [58].

The Psychological Well-Being Index (PWBI) analyzes psychological health and well-being through anxiety, depression, well-being, vitality, self-control, and general health. The authors describe self-control as mastery of the environment. It consists of 22 items and has been used in clinical trials with patients. Specifically, in the study analyzed, the participants were patients of the center and former patients who currently practice sports. Its reliability is high, 0.94. It has recently been used in the hospital context with nurses [59].

Two studies analyzed the Domain dimension to measure athletes’ perceived strengths in controlling and predicting their lives, all in relation to the physical and social environment [14,44]. This dimension is measured through seven items. The scale has an acceptable reliability of 0.70. The scale has recently been used alongside life satisfaction in the field of sociology [60].

Other scales have also analyzed psychological well-being, and the most widely used has been WHOQoL-BREF [47]. It has its origin in the WHOQOL-100 questionnaire, which is a reduced version composed of 26 items and measures four dimensions related to quality of life and well-being at the physical, psychological, social, and environmental levels. Its reliability index is 0.89. In the search, we also found an adaptation of the WHOQoL-BREF questionnaire for people with physical disabilities called WHOQOL-DIS [51]. Its reliability index is 0.85. The WHOQOL-BREF scale has recently been used to analyze QOL in athletes [61] and the WHOQOL-DIS scale in people with spinal cord injury [62].

Table 4 shows the most analyzed dimensions. Autonomy is one of the most significant dimensions in the context of people with physical disabilities, specifically the degree of dependence, and the origin of the disability significantly influences their psychological well-being. In the study of [37], we have analyzed that the higher the degree of independence, the higher the PWB. Regarding the origin of disability studies, they show that people with acquired disability have greater autonomy [19,63,64]. The authors state that athletes with acquired disability have had to face adverse situations and have had to adapt and develop new skills. That affirms this theory and shows that people with acquired disability, who were patients who practiced physical exercise, had higher levels of psychological and subjective well-being compared to people with a congenital disability [65]. Regarding sports practice, subjects who practice sports have greater autonomy [35] and, consequently, higher PWB. One of the studies points out that the lack of autonomy is one of the main causes of low PWB values [34]. Autonomy, despite being an essential factor in the well-being of people with disabilities, in the study of [34], is the dimension with the lowest score of the total Ryff dimensions. It would be interesting to consider the degree of dependence of athletes with disabilities to differentiate the profile and needs of people.

The second dimension analyzes the mastery of the environment and has been the most evaluated by the authors. On this occasion, the origin of the disability also influences the assessment of the subjects. Specifically, people with congenital disability have a lower mastery of the environment since they have difficulties from the beginning of their lives in managing situations and generally have not had to adapt to a new situation of dependence compared to people with acquired disabilities [19,63,64]. Regarding participation in sports activities, the greater the practice and the higher the level of competition, the greater the mastery of the environment [32,36].

In relation to mastery and sense of control, one of the studies analyzes the self-efficacy variable and shows a statistically significant relationship with PWB [31]. Self-efficacy is considered an indispensable element in predicting the psychological abilities and strengths of athletes [66]. Along the same line, one of the studies highlights the importance of deepening psychological skills, such as resilience and optimism among others to understand the psychological well-being of athletes [36]. Three of the recently mentioned variables, self-efficacy, resilience, and optimism, together with hope, form a single construct called psychological capital and have recently been adapted to the sports context to comprehensively assess the psychological strengths of athletes with a reliability value of 0.89 [67].

In one of the studios, personal growth was the dimension most valued by athletes, and in their study, they also analyzed subjective vitality as an indicator of well-being obtaining favorable results [33]. This domain is fundamental for the PWB as it refers to the person’s ability to meet goals and develop as a person.

With respect to social bonds, sport is an essential tool in social-inclusion processes, and, specifically, this dimension focuses on analyzing people’s ability to develop and maintain satisfactory social relationships [15,67,68]. Studies advocate that social relationships are closely related to psychological well-being and quality of life of people, improving self-esteem and reducing stress [69]. The social ties dimension includes aspects such as social support, a subjective perception of the quality, and support received by the environment [70]. In this line, the studies defend the need to establish social bonds to obtain positive PWB results and expose that the lack of security about the person’s qualities can reduce PWB [36].

Regarding the projects dimension, athletes value the dimension of projects and social ties in the same way [34]. They exceed the valuations in the dimensions of mastery and autonomy, which provides more relevance to the projects domain. In addition, it analyzes the aspirations and goals of athletes with physical disabilities, which is a key aspect in determining the well-being of athletes and, in turn, ensures continuity in sports practice [71].

Acceptance has been analyzed as a dimension of the PWB in two of the articles reviewed, but the authors do not show significant results with respect to this dimension. Therefore, it would be interesting to identify mental toughness following the contribution of the studies and, in turn, analyze the perceived barriers to understand the results [37]. 

All the articles show a positive relationship between sports practice and PWB. Recent literature shows that sport improves the dimensions implicit in the PWB of Ryff’s model, including self-sufficiency, independence, achievement of goals, improvement of growth, and personal development [72,73].

In our review, the main significant difference is found between athletes and non-athletes, and among athletes, it is observed that those who practice more sports show higher PWB values [19,24,32,34,35,36,37,74]. Among athletes, it is also observed that the higher the level of competition, the higher the PWB, a factor that in turn influences the improvement of sports performance by the development of athletes’ abilities [19,32]. In this sense, there is more and more psychological programming with the aim of improving sports performance [75,76,77,78]. The studies confirmed that PWB can be improved [55].

## 5. Conclusions

From the analysis performed on the studies in this systematic review, we can conclude that sports practice has a positive relationship with the psychological well-being of athletes with physical disabilities. The 13 studies have shown positive relationships, and only two studies have not found statistically significant differences in their specific dimension of psychological well-being, but only in its totality. The most relevant dimensions were mastery of the environment and autonomy, with acceptance having the least impact. The main limitations were the paucity of information, the small sample sizes used, and the lack of studies focused on under-age athletes. To date, no research has been found that analyses psychological well-being in under-age athletes. The scarcity of variables analyzed has limited the results. It is recommended that future research includes more variables, such as the type of sport, level of competition, experience, disability, frequency of practice, and level of dependence. In addition, this study suggests the need to expand research on the psychological well-being of young athletes with disabilities, with the aim of enriching the existing literature and deepening knowledge in this area to develop intervention programs aimed at improving their psychological well-being. To achieve this, it would be essential to train the professionals who accompany athletes since ignoring psychological resources could lead to sports abandonment and hinder the inclusion of people with disabilities. At the same time, this study reinforces the need to use specific tools that measure PWB, and after the analysis, we highlight the BIEPS scale for its novel results and also include the dimension of autonomy, which has greater reliability and a specific version for young people. In conclusion, we consider that these results are important to increase knowledge about the influence of sport on people with physical disabilities by contributing to the improvement of their health, personal development, and inclusion in society.

## Figures and Tables

**Table 1:** Search strategy.

Articles Found				
	Pubmed	Wos	Scopus	Sportdiscus
1-“Psychological well-being” OR “Psychological well-being” AND sport AND disabil *	6	554	106	49
2-Physic * AND disab * AND sport AND state of mind	1	0	1	0
3-Physic * AND disab * AND sport AND anxiety	21	31	82	32
4-Physic * AND disab * AND sport AND self-esteem	19	32	0	20
5-Physic * AND disab * AND sport AND environment	40	83	186	74
6-Physic * AND disab * AND sport AND self-perception	5	14	28	14
7-Physic * AND disab * AND sport AND quality of life	110	170	7	170
8-Physic * AND disab * AND sport AND social relations *	4	8	17	10
9-Physic * AND disab * AND sport AND psychological disorders	0	1	2	2
10-Physic * AND disab * AND sport AND depression	22	42	114	33
11-Physic * AND disab * AND sport AND subjective vitality	0	0	2	0
Total without duplicates	1342			

Note: The use of the asterisk (*) allows for more complete searches, capturing different variations of the root term, such as “disability”, “disabilities”, “disabled”, among others.

## Data Availability

The data that support the findings of this study are available from the corresponding author upon reasonable request.
